# (1*S*,3*R*,8*R*,11*S*)-2,2-Dichloro-3,7,7,10-tetra­methyl­tricyclo­[6.4.0.0^1,3^]dodec-9-en-11-ol

**DOI:** 10.1107/S1600536811004788

**Published:** 2011-02-12

**Authors:** Ahmed Benharref, Essêdiya Lassaba, Daniel Avignant, Abdelghani Oudahmane, Moha Berraho

**Affiliations:** a Laboratoire de Chimie Biomoléculaires, Substances Naturelles et Réactivité, URAC16, Faculté des Sciences, Semlalia, BP 2390 Bd My Abdellah, 40000 Marrakech, Morocco; b Université Blaise Pascal, Laboratoire des Matériaux Inorganiques, UMR CNRS 6002, 24 Avenue des Landais, 63177 Aubière, France

## Abstract

The title compound, C_16_H_24_Cl_2_O, was synthesized from β-himachalene (3,5,5,9-tetra­methyl-2,4a,5,6,7,8-hexa­hydro-1*H*-benzocyclo­heptene), which was isolated from essential oil of the Atlas cedar (*Cedrus atlantica*). The two fused rings exhibit different conformations: the six-membered ring has a screw-boat conformation, while the seven-membered ring displays a boat conformation. The dihedral angle between the two rings is 56.56 (18)°. In the crystal, mol­ecules aggregate into supra­molecular chains along the *c* axis mediated by O—H⋯O hydrogen bonds.

## Related literature

For the isolation of β-himachalene, see: Joseph & Dev (1968 ▶); Plattier & Teisseire (1974 ▶). For the reactivity of this sesquiterpene, see: Lassaba *et al.* (1998 ▶); Chekroun *et al.* (2000 ▶); El Jamili *et al.* (2002 ▶); Sbai *et al.* (2002 ▶); Dakir *et al.* (2004 ▶). For its biological activity, see: Daoubi *et al.* (2004 ▶). For conformational analysis, see: Cremer & Pople (1975 ▶).
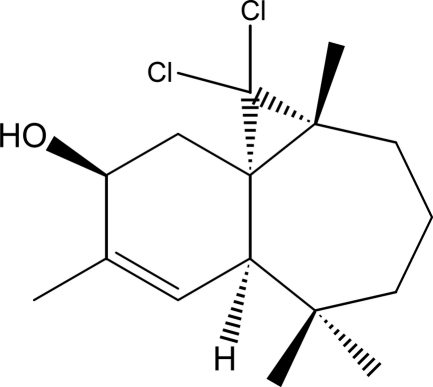


## Experimental

### 

#### Crystal data

C_16_H_24_Cl_2_O
*M* *_r_* = 303.25
Trigonal, 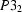

*a* = 13.2323 (13) Å
*c* = 7.9807 (8) Å
*V* = 1210.2 (2) Å^3^
*Z* = 3
Mo *K*α radiation
μ = 0.39 mm^−1^
*T* = 298 K
0.41 × 0.33 × 0.26 mm

#### Data collection

Bruker APEXII CCD diffractometer
8123 measured reflections
3135 independent reflections
2995 reflections with *I* > 2σ(*I*)
*R*_int_ = 0.019

#### Refinement

*R*[*F* ^2^ > 2σ(*F* ^2^)] = 0.045
*wR*(*F* ^2^) = 0.126
*S* = 1.09
3135 reflections
180 parameters
1 restraint
H-atom parameters constrained
Δρ_max_ = 0.52 e Å^−3^
Δρ_min_ = −0.33 e Å^−3^
Absolute structure: Flack (1983 ▶), 1940 Friedel pairs
Flack parameter: −0.11 (7)

### 

Data collection: *APEX2* (Bruker, 2009 ▶); cell refinement: *SAINT* (Bruker, 2009 ▶); data reduction: *SAINT*; program(s) used to solve structure: *SHELXS97* (Sheldrick, 2008 ▶); program(s) used to refine structure: *SHELXL97* (Sheldrick, 2008 ▶); molecular graphics: *ORTEP-3 for Windows* (Farrugia, 1997 ▶); software used to prepare material for publication: *WinGX* (Farrugia, 1999 ▶).

## Supplementary Material

Crystal structure: contains datablocks I, global. DOI: 10.1107/S1600536811004788/tk2714sup1.cif

Structure factors: contains datablocks I. DOI: 10.1107/S1600536811004788/tk2714Isup2.hkl

Additional supplementary materials:  crystallographic information; 3D view; checkCIF report

## Figures and Tables

**Table 1:** Hydrogen-bond geometry (Å, °)

*D*—H⋯*A*	*D*—H	H⋯*A*	*D*⋯*A*	*D*—H⋯*A*
O1—H1⋯O1^i^	0.82	2.10	2.853 (4)	153

Symmetry code: (i) 
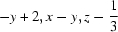
.

**Fig. 1.**
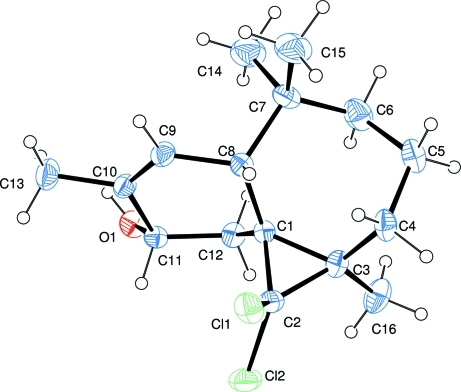
Molecular structure of the title compound with the atom-labelling scheme. Displacement ellipsoids are drawn at the 30% probability level. H atoms are represented as small spheres of arbitrary radii.

**Fig. 2.**
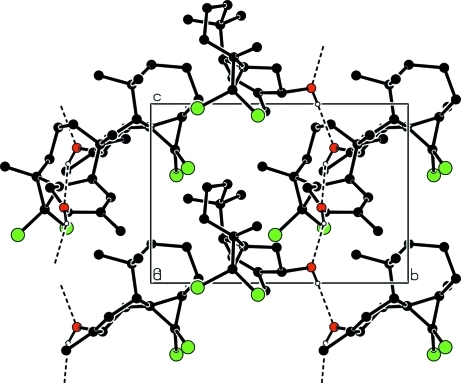
Partial packing diagram showing the O—H···O interactions (dashed lines) and the formation of supramolecular chains parallel to the c axis. H atoms not involved in hydrogen bonding have been omitted for clarity.

## supplementary crystallographic information

### Comment

The bicyclic sesquiterpene β-himachalene is the main constituent of the essential oil of the Atlas cedar (*Cedrus atlantica*) (Joseph & Dev, 1968; Plattier & Teisseire, 1974). The reactivity of this sesquiterpene and its derivatives has been studied extensively by our team in order to prepare new products having biological proprieties (Lassaba *et al.*, 1998; Chekroun *et al.*, 2000; El Jamili *et al.*, 2002; Sbai *et al.*, 2002; Dakir *et al.*, 2004). Indeed, these compounds were tested, using the food poisoning technique, for their potential antifungal activity against the phytopathogen *Botrytis cinerea* (Daoubi *et al.*, 2004).

The action of one equivalent of dichlorocarbene, generated *in situ* from chloroform in the presence of sodium hydroxide as base and n-benzyltriethylammonium chloride as catalyst, on β-himachalene produces only (1*S*,3*R*,8*R*)-2,2-dichloro-3,7,7,10- tetramethyltricyclo[6.4.0.0^1,3^]dodec-9-ene (El Jamili *et al.*, 2002). Treatment of the latter compound with two equivalents of *N*-bromosuccinimide gives (1*S*, 3*R*, 8*R*, 11S)-2,2-dichloro -3,7,7,10-tetralethyltricyclo[6.4.0.0^1,3^]dodec-9-en-11-ol in a very low yield (5%), along with other products. The structure of this new product was determined by NMR (^1^H & ^13^C) spectral analysis and mass spectroscopy, and confirmed by a crystallographic study, reported herein.

The molecule is built up from two fused six-membered and seven-membered rings (Fig. 1). The six-membered ring has a screw boat conformation, as indicated by the total puckering amplitude QT = 0.480 (3) Å and spherical polar angle θ = 130.6 (4) ° with φ = 151.5 (5) °, whereas the seven-membered ring displays a boat conformation with QT = 1.1449 (30) Å, θ_2_ = 88.29 (15) °, φ_2_ = -47.13 (14) ° and φ_3_ =-144.24 (5) ° (Cremer & Pople, 1975). In the crystal structure, molecules are linked into supramolecular chains (Fig. 2) running along the *c* axis by O—H···O hydrogen bonds (Table 1). Owing to the presence of Cl atoms, the absolute configuration could be fully confirmed, as C1(*S*), C3(*R*), C8(*R*) and C11(*S*).

### Experimental

In a reactor containing a solution of (1*S*, 3*R*, 8*R*)-2,2-dichloro-3,7,7,10 tetramethyltricyclo [6.4.0.0^1,3^] dodec-9-ene (1 g, 3.48 mmol) in 50 ml of tetrahydrofuran and water (THF/H_2_O) (4:1) cooled to 273 K and kept in the dark, was added in small portions 1.23 g (6.96 mmol) of *N*-bromosccinimide. The reaction mixture was left stirring for 1 h, after which 20 ml of a saturated solution of NaHCO_3_ was added. Subsequently, the extraction was performed three times with diethyl ether (3 x 20 ml). The organic extracts were dried over Na_2_SO_4_, filtered, concentrated, and chromatographed. The title compound, (1*S*, 3*R*, 8*R*, 11*S*,)-2,2-dichloro-3,7,7,10-tetralethyltricyclo [6.4.0.0^1,3^] dodec-9-by-11-ol was obtained with in a yield of 5% and was recrystallized its pentane solution.

### Refinement

All H atoms were fixed geometrically and treated as riding with O—H = 0.82 Å and C—H = 0.93 (ethylene), 0.96 Å (methyl), 0.97 Å (methylene) and 0.98Å (methine), and with *U*_iso_(H) = 1.2U_eq_ (ethylene, methylene, methine) or *U*_iso_(H) = 1.5*U*_eq_ (O, methyl).

### Crystal data

**Table:**

C_16_H_24_Cl_2_O	*D*_x_ = 1.244 Mg m^−^^3^
*M**_r_* = 303.25	Mo *K*α radiation, λ = 0.71073 Å
Trigonal, *P*3_2_	Cell parameters from 8123 reflections
Hall symbol: P 32	θ = 4–26.4°
*a* = 13.2323 (13) Å	µ = 0.39 mm^−^^1^
*c* = 7.9807 (8) Å	*T* = 298 K
*V* = 1210.2 (2) Å^3^	Prism, colourless
*Z* = 3	0.41 × 0.33 × 0.26 mm
*F*(000) = 486	

### Data collection

**Table:**

Bruker APEXII CCD diffractometer	2995 reflections with *I* > 2σ(*I*)
Radiation source: fine-focus sealed tube	*R*_int_ = 0.019
graphite	θ_max_ = 26.4°, θ_min_ = 4.0°
ω and φ scans	*h* = −15→16
8123 measured reflections	*k* = −14→16
3135 independent reflections	*l* = −9→9

### Refinement

**Table:**

Refinement on *F*^2^	Secondary atom site location: difference Fourier map
Least-squares matrix: full	Hydrogen site location: inferred from neighbouring sites
*R*[*F*^2^ > 2σ(*F*^2^)] = 0.045	H-atom parameters constrained
*wR*(*F*^2^) = 0.126	*w* = 1/[σ^2^(*F*_o_^2^) + (0.0761*P*)^2^ + 0.4405*P*] where *P* = (*F*_o_^2^ + 2*F*_c_^2^)/3
*S* = 1.09	(Δ/σ)_max_ < 0.001
3135 reflections	Δρ_max_ = 0.52 e Å^−^^3^
180 parameters	Δρ_min_ = −0.33 e Å^−^^3^
1 restraint	Absolute structure: Flack & Bernardinelli (2000), 1940 Friedel pairs
Primary atom site location: structure-invariant direct methods	Flack parameter: −0.11 (7)

### Special details

**Table:**

Geometry. All s.u.'s (except the s.u. in the dihedral angle between two l.s. planes) are estimated using the full covariance matrix. The cell s.u.'s are taken into account individually in the estimation of s.u.'s in distances, angles and torsion angles; correlations between s.u.'s in cell parameters are only used when they are defined by crystal symmetry. An approximate (isotropic) treatment of cell s.u.'s is used for estimating s.u.'s involving l.s. planes.

### Fractional atomic coordinates and isotropic or equivalent isotropic displacement parameters (Å^2^)

**Table:**

	*x*	*y*	*z*	*U*_iso_*/*U*_eq_	
C1	1.0043 (2)	0.6432 (2)	0.5388 (3)	0.0304 (5)	
C2	0.9057 (2)	0.5880 (2)	0.4113 (4)	0.0370 (5)	
C3	0.8790 (2)	0.5564 (2)	0.5938 (4)	0.0392 (6)	
C4	0.8143 (3)	0.6069 (3)	0.6915 (4)	0.0464 (7)	
H4A	0.8228	0.6749	0.6329	0.056*	
H4B	0.7319	0.5493	0.6955	0.056*	
C5	0.8600 (3)	0.6411 (3)	0.8682 (5)	0.0541 (8)	
H5A	0.8234	0.5720	0.9384	0.065*	
H5B	0.8370	0.6953	0.9106	0.065*	
C6	0.9973 (3)	0.6991 (4)	0.8854 (5)	0.0591 (9)	
H6A	1.0178	0.7232	1.0010	0.071*	
H6B	1.0175	0.6394	0.8633	0.071*	
C7	1.0733 (2)	0.8032 (3)	0.7746 (4)	0.0426 (6)	
C8	1.0545 (2)	0.7728 (2)	0.5810 (3)	0.0308 (5)	
H8	0.9966	0.7933	0.5426	0.041 (8)*	
C9	1.1625 (2)	0.8449 (2)	0.4781 (4)	0.0394 (6)	
H9	1.1895	0.9246	0.4721	0.039 (8)*	
C10	1.2228 (2)	0.8058 (2)	0.3951 (4)	0.0367 (5)	
C11	1.1869 (2)	0.6778 (2)	0.3958 (3)	0.0333 (5)	
H11	1.1523	0.6443	0.2867	0.029 (7)*	
C12	1.0980 (2)	0.6089 (2)	0.5320 (4)	0.0364 (5)	
H12A	1.0624	0.5260	0.5090	0.044*	
H12B	1.1371	0.6242	0.6396	0.044*	
C13	1.3285 (3)	0.8828 (3)	0.2905 (5)	0.0549 (8)	
H13A	1.3407	0.9607	0.2893	0.082*	
H13B	1.3164	0.8532	0.1780	0.082*	
H13C	1.3957	0.8837	0.3373	0.082*	
C14	0.8465 (3)	0.4338 (3)	0.6503 (6)	0.0633 (10)	
H14A	0.7653	0.3913	0.6796	0.095*	
H14B	0.8927	0.4386	0.7459	0.095*	
H14C	0.8611	0.3943	0.5608	0.095*	
C15	1.0481 (4)	0.9031 (4)	0.8042 (6)	0.0718 (11)	
H15A	0.9719	0.8811	0.7621	0.108*	
H15B	1.1055	0.9718	0.7469	0.108*	
H15C	1.0512	0.9188	0.9221	0.108*	
C16	1.2019 (4)	0.8507 (4)	0.8255 (6)	0.0730 (11)	
H16A	1.2122	0.8740	0.9410	0.109*	
H16B	1.2515	0.9168	0.7568	0.109*	
H16C	1.2219	0.7909	0.8102	0.109*	
O1	1.28683 (18)	0.6633 (2)	0.4212 (3)	0.0453 (5)	
H1	1.3211	0.6727	0.3319	0.068*	
Cl1	0.85533 (6)	0.67322 (7)	0.30875 (9)	0.0497 (2)	
Cl2	0.90015 (7)	0.48357 (7)	0.26866 (11)	0.0605 (2)	

### Atomic displacement parameters (Å^2^)

**Table:**

	*U*^11^	*U*^22^	*U*^33^	*U*^12^	*U*^13^	*U*^23^
C1	0.0314 (11)	0.0304 (11)	0.0315 (12)	0.0170 (10)	0.0015 (9)	0.0018 (9)
C2	0.0353 (12)	0.0343 (12)	0.0391 (14)	0.0157 (10)	−0.0032 (10)	−0.0049 (10)
C3	0.0350 (13)	0.0374 (13)	0.0431 (15)	0.0164 (11)	0.0042 (11)	0.0079 (11)
C4	0.0365 (14)	0.0604 (18)	0.0436 (16)	0.0253 (13)	0.0107 (11)	0.0114 (13)
C5	0.0556 (19)	0.069 (2)	0.0418 (16)	0.0344 (17)	0.0144 (14)	0.0117 (15)
C6	0.065 (2)	0.079 (2)	0.0401 (17)	0.0407 (19)	−0.0012 (14)	0.0047 (16)
C7	0.0435 (14)	0.0539 (16)	0.0364 (14)	0.0289 (13)	−0.0053 (11)	−0.0123 (12)
C8	0.0306 (11)	0.0319 (12)	0.0351 (12)	0.0197 (10)	0.0012 (9)	−0.0015 (9)
C9	0.0409 (14)	0.0287 (12)	0.0461 (16)	0.0155 (10)	0.0048 (11)	0.0006 (10)
C10	0.0320 (12)	0.0339 (13)	0.0409 (14)	0.0141 (10)	0.0025 (10)	−0.0003 (10)
C11	0.0327 (12)	0.0367 (12)	0.0365 (14)	0.0218 (10)	−0.0017 (9)	−0.0045 (10)
C12	0.0369 (12)	0.0343 (12)	0.0451 (14)	0.0230 (11)	0.0031 (10)	0.0023 (10)
C13	0.0452 (16)	0.0493 (17)	0.064 (2)	0.0190 (14)	0.0200 (15)	0.0072 (14)
C14	0.0562 (19)	0.0404 (16)	0.082 (3)	0.0158 (15)	0.0123 (17)	0.0191 (16)
C15	0.078 (3)	0.071 (2)	0.078 (3)	0.046 (2)	0.002 (2)	−0.023 (2)
C16	0.058 (2)	0.087 (3)	0.069 (3)	0.033 (2)	−0.0150 (19)	−0.023 (2)
O1	0.0410 (10)	0.0614 (13)	0.0479 (12)	0.0365 (10)	0.0009 (8)	−0.0025 (9)
Cl1	0.0471 (4)	0.0680 (5)	0.0384 (3)	0.0321 (4)	−0.0065 (3)	0.0047 (3)
Cl2	0.0541 (4)	0.0536 (4)	0.0643 (5)	0.0199 (4)	−0.0071 (4)	−0.0266 (4)

### Geometric parameters (Å, °)

**Table:**

C1—C12	1.521 (3)	C9—C10	1.326 (4)
C1—C2	1.522 (3)	C9—H9	0.9300
C1—C3	1.534 (3)	C10—C13	1.506 (4)
C1—C8	1.535 (3)	C10—C11	1.513 (4)
C2—C3	1.507 (4)	C11—O1	1.442 (3)
C2—Cl2	1.764 (3)	C11—C12	1.524 (4)
C2—Cl1	1.771 (3)	C11—H11	0.9800
C3—C14	1.524 (4)	C12—H12A	0.9700
C3—C4	1.535 (4)	C12—H12B	0.9700
C4—C5	1.512 (5)	C13—H13A	0.9600
C4—H4A	0.9700	C13—H13B	0.9600
C4—H4B	0.9700	C13—H13C	0.9600
C5—C6	1.584 (5)	C14—H14A	0.9600
C5—H5A	0.9700	C14—H14B	0.9600
C5—H5B	0.9700	C14—H14C	0.9600
C6—C7	1.518 (5)	C15—H15A	0.9600
C6—H6A	0.9700	C15—H15B	0.9600
C6—H6B	0.9700	C15—H15C	0.9600
C7—C15	1.534 (5)	C16—H16A	0.9600
C7—C16	1.545 (5)	C16—H16B	0.9600
C7—C8	1.585 (4)	C16—H16C	0.9600
C8—C9	1.505 (3)	O1—H1	0.8200
C8—H8	0.9800		
			
C12—C1—C2	117.7 (2)	C1—C8—H8	106.2
C12—C1—C3	121.6 (2)	C7—C8—H8	106.2
C2—C1—C3	59.08 (17)	C10—C9—C8	126.2 (2)
C12—C1—C8	112.3 (2)	C10—C9—H9	116.9
C2—C1—C8	118.1 (2)	C8—C9—H9	116.9
C3—C1—C8	118.4 (2)	C9—C10—C13	123.3 (3)
C3—C2—C1	60.86 (17)	C9—C10—C11	121.5 (2)
C3—C2—Cl2	119.6 (2)	C13—C10—C11	115.2 (2)
C1—C2—Cl2	119.77 (19)	O1—C11—C10	110.7 (2)
C3—C2—Cl1	120.9 (2)	O1—C11—C12	107.7 (2)
C1—C2—Cl1	120.59 (18)	C10—C11—C12	112.9 (2)
Cl2—C2—Cl1	108.61 (15)	O1—C11—H11	108.5
C2—C3—C14	118.9 (3)	C10—C11—H11	108.5
C2—C3—C1	60.06 (16)	C12—C11—H11	108.5
C14—C3—C1	120.3 (3)	C1—C12—C11	110.3 (2)
C2—C3—C4	118.3 (2)	C1—C12—H12A	109.6
C14—C3—C4	113.0 (3)	C11—C12—H12A	109.6
C1—C3—C4	116.7 (2)	C1—C12—H12B	109.6
C5—C4—C3	112.2 (3)	C11—C12—H12B	109.6
C5—C4—H4A	109.2	H12A—C12—H12B	108.1
C3—C4—H4A	109.2	C10—C13—H13A	109.5
C5—C4—H4B	109.2	C10—C13—H13B	109.5
C3—C4—H4B	109.2	H13A—C13—H13B	109.5
H4A—C4—H4B	107.9	C10—C13—H13C	109.5
C4—C5—C6	114.6 (3)	H13A—C13—H13C	109.5
C4—C5—H5A	108.6	H13B—C13—H13C	109.5
C6—C5—H5A	108.6	C3—C14—H14A	109.5
C4—C5—H5B	108.6	C3—C14—H14B	109.5
C6—C5—H5B	108.6	H14A—C14—H14B	109.5
H5A—C5—H5B	107.6	C3—C14—H14C	109.5
C7—C6—C5	118.0 (3)	H14A—C14—H14C	109.5
C7—C6—H6A	107.8	H14B—C14—H14C	109.5
C5—C6—H6A	107.8	C7—C15—H15A	109.5
C7—C6—H6B	107.8	C7—C15—H15B	109.5
C5—C6—H6B	107.8	H15A—C15—H15B	109.5
H6A—C6—H6B	107.2	C7—C15—H15C	109.5
C6—C7—C15	111.2 (3)	H15A—C15—H15C	109.5
C6—C7—C16	108.2 (3)	H15B—C15—H15C	109.5
C15—C7—C16	106.2 (3)	C7—C16—H16A	109.5
C6—C7—C8	112.8 (2)	C7—C16—H16B	109.5
C15—C7—C8	107.1 (3)	H16A—C16—H16B	109.5
C16—C7—C8	111.1 (3)	C7—C16—H16C	109.5
C9—C8—C1	109.4 (2)	H16A—C16—H16C	109.5
C9—C8—C7	113.1 (2)	H16B—C16—H16C	109.5
C1—C8—C7	115.0 (2)	C11—O1—H1	109.5
C9—C8—H8	106.2		

### Hydrogen-bond geometry (Å, °)

**Table:**

*D*—H···*A*	*D*—H	H···*A*	*D*···*A*	*D*—H···*A*
O1—H1···O1^i^	0.82	2.10	2.853 (4)	153

Symmetry codes: (i) −*y*+2, *x*−*y*, *z*−1/3.
